# Rheological Behavior of Blends of Metallocene Catalyzed Long-Chain Branched Polyethylenes. Part I: Shear Rheological and Thermorheological Behavior

**DOI:** 10.3390/polym13030328

**Published:** 2021-01-20

**Authors:** Chuangbi Chen, Mehdihasan I. Shekh, Shuming Cui, Florian J. Stadler

**Affiliations:** College of Materials Science and Engineering, Shenzhen Key Laboratory of Polymer Science and Technology, Guangdong Research Center for Interfacial Engineering of Functional Materials, Nanshan District Key Laboratory for Biopolymers and Safety Evaluation, Shenzhen University, Shenzhen 518055, China; 1810342023@email.szu.edu.cn (C.C.); mehdi.shekh3@yahoo.com (M.I.S.); 1910342023@email.szu.edu.cn (S.C.)

**Keywords:** polyethylenes, blends, long-chain branches, thermorheological complexity, activation energy spectrum

## Abstract

Long-chain branched metallocene-catalyzed high-density polyethylenes (LCB-mHDPE) were solution blended to obtain blends with varying degrees of branching. A high molecular LCB-mHDPE was mixed with low molecular LCB-mHDPE at varying concentrations. The rheological behavior of those low molecular LCB-mHDPE is similar but their molar mass and molar mass distribution are significantly different. Those blends were characterized rheologically to study the effects of concentration, molar mass distribution, and long-chain branching level of the low molecular LCB-mHDPE. Owing to the ultra-long relaxation times of the high molecular LCB-mHDPE, the blends exhibited a clearly more long-chain branched behavior than the base materials. The thermorheological complexity analysis showed an apparent increase in the activation energies *E_a_* determined from *G′*, *G″*, and especially δ. *E_a_*(δ), which for LCB-mHDPE is a peak function, turned out to produce even more pronounced peaks than observed for LCB-mPE with narrow molar mass distribution and also LCB-mPE with broader molar mass distribution. Thus, it is possible to estimate the molar mass distribution from the details of the thermorheological complexity.

## 1. Introduction

Polyolefins account for ca. 50% of the synthetic polymers produced worldwide, mainly used for packaging, cable insulation, and household goods. The most commonly used polyolefin is polyethylene (PE), chemically correctly also known as polyethene, comprising ca. 30% of the world polymer production, mainly applied for film manufacturing and cable sheathing. Both processes include strong elongational deformations, for which strain hardening has proven to be essential for obtaining a stable process and a homogeneous film or layer thickness [1,2].

The strain hardening for classical long-chain branched polyethylene, the well-known low-density polyethylene (LDPE), is established to be very good, while for the Ziegler-Natta polyethylene (linear low density (ZN-LLDPE) or high density (ZN-HDPE)) usually no strain hardening, at least at high Hencky strain rates [3] $\dot{\epsilon }\;$ is found [1,2,4,5]. LCB-mPE shows weak strain hardening, predominantly at low strain rates $\dot{\epsilon }\;$ making these materials potentially better processable. However, in general the strain hardening of LCB-mPE is not very high, so that the processing behavior is still not entirely on par with LDPE. The literature on strain hardening of PE is very broad and will be discussed in detail in part II or this paper series. To provide a simplified summary, the more LCBs are in a material (e.g., per 1000 C), the more strain-hardening is the material. As LCB-mPE has typically about factor 5–10 less long-chain branches than LDPE, their strain hardening level is not as high.

In the past, attempts have been made to blend LDPE with LLDPE (ZN-LLDPE, mostly), which has led to a mix of both properties, i.e., the processing improved in comparison to LLDPE, while some decrease of its mechanical advantages have to be taken into account.

In the field of analytical characterization of polymers with respect to their branching topography, many attempts were made to characterize the branching structures, which, however, has always suffered from the ambiguity that the effects of long-chain branching and molar mass distribution are very similar to each other in shear rheology so that easy approaches to characterize samples with respect to their understanding their long-chain branching topography will not deliver reliable results for polydisperse materials. As is well known, the relation between zero shear-rate viscosity *η*_0_ and weight-average molar mass *M_w_* (*η*_0_ ~ *M_w_*^3.4^) for linear polymers was found to be molar mass distribution (MMD) independent [6,7,8]. However, this relation requires the determination of *M_w_* by analytical means, and when taking a ±5% error of *M_w_*-determination into account, only deviations by more than 20% with respect to *η*_0_ can be considered reliable. Further, for many materials determining an accurate *M_w_* is not straightforward. Furthermore, especially for samples with very broad molar mass distribution, the determination of the true *M_w_* is far from obvious, so that it is questioned, whether for some “exotic” molar mass distributions the relation *η*_0_~*M_w_*^3.4^ would have to be amended with some MMD-dependent terms [9,10,11,12], although the authors believe that it is more likely that this is not the case.

However, another rheological quantity is not dependent on the molar mass distribution, while it reacts to long-chain branching–the thermorheological behavior, as long as it follows an Arrhenius temperature dependence [13,14,15,16,17,18,19]. For materials following a WLF-temperature dependence, long-chain branching does not have an influence on the temperature dependence [20].

As already pointed out by Carella, Gotro, and Graessley in 1986 [21], the increase of Arrhenius activation energy *E_a_* is accompanied by thermorheological complexity, i.e., the failure of the time-temperature superposition. This can be explained by long-chain branching related relaxation processes with a higher activation energy occurring relatively close on the relaxation time scale to “normal” relaxation processes for linear chains with the normal activation energy for the polymer under investigation (for HDPE: *E_a_* = 27 kJ/mol. The consequence is that the relative importance of these processes for the rheological data as a function of, e.g., angular frequency *ω* varies, which leads to a non-constant *E_a_* and, thus, to thermorheological complexity, as the shape of the rheological functions varies due to the temperature dependence of the shape of the relaxation (or retardation) spectrum [22]. Thermorheological complexity was previously found for long-chain branched polyethylene (discussed below), polypropylene [23,24,25,26], hydrogenated polybutadiene [16,21], fluoro-polymers [27], and polylactides [28]. The authors are not aware of any reliable publications, where long-chain branching in Arrhenius-type materials does not lead to an increase in *E_a_* and to thermorheological complexity. Changes in *E_a_* can also be caused by changes in tacticity (e.g., isotactic polypropylene has a lower *E_a_* than syndiotactic polypropylene [25,29]) and copolymerization (e.g., ethylene-octene copolymers typically have *E_a_* = 29–33 kJ/mol [16,30,31,32,33,34,35,36]), but those do not lead to thermorheological complexity and will not be discussed in this paper.

Thus, to analyze the temperature dependence of LCB-materials with an Arrhenius temperature dependence, the simplest possible way would be to ignore the thermorheological complexity and make the best global fit (using one shift factor a_T_ per temperature), which would be scientifically incorrect and would lead to ambiguous results [36]. e.g., the results would depend on the frequency range for which they were calculated. First attempts for a proper analysis of the thermorheological complexity were presented by Carella et al. [21] and Laun [37], who did not analyze the temperature dependence globally but locally, namely by determining the shift factors from specific values of *G”* and the shear stress σ_21_, respectively. For LCB-mPE, the thermorheological complexity was first described by Wood-Adams and Costeux through determining activation energies derived from storage modulus *G′*, loss modulus *G″*, the relaxation spectrum, and *η*_0_ [15]. Based on these initial reports, the authors systematically analyzed the thermorheological complexity by determining the rheological spectra with high precision methods [38,39,40,41,42] and shifting them slicewise [22,31,41]. However, while shifting the relaxation spectrum is the fundamentally most meaningful way of analyzing such a complicated behavior, it is very cumbersome to do so, as the calculation of a rheological spectrum is a highly difficult undertaking, being easily influenced by various artefacts.

Instead, research has shown that shifting *G′*, *G″* and phase angle *δ* can provide insight into different aspects of the thermorheological complexity of branched LCB-mPE [22,42]. However, at this point, the determination still suffered from the fact that the determination of the shift factors as a function of the rheological quantity (e.g., *G′*, *δ*) still had to be done manually. For this reason, the authors developed a protocol for determining the shift factors by a numerical procedure and even more precisely and classified the resulting types of thermorheological complexity [43].

Bai et al. [44] and Shen et al. [45] blended low and high molecular PE with *M_w_*-ratios around 2.5 and 10 and performed a basic rheological characterization, respectively. They found a power-law mixing law; however, based on their data, their materials are linear. Chen et al. [46] made blends of LDPE, LLDPE with an ultrahigh molecular PE and found melt-miscibility but not in solid state. They found thermorheological complexity but did not evaluate it. Chaudhuri et al. [47] melt blended normal HDPE with ultrahigh molecular PE, which was synthesized in a special way that it was unentangled despite the high molar mass in order to facilitate melt blending. This resulted in superior blend quality and improved strain hardening.

The thermorheology of polymer blends was investigated for ZN-LLDPE/LDPE and mLLDPE/mVLDPE (very low-density PE) blends by Dordinejad et al. [18,48]. In the case of mLLDPE/mVLDPE-blends, the absence of long-chain branches leads to essentially thermorheologically simple samples [48]. However, the LDPE/LLDPE blends [18] show a clear thermorheological complexity, which is systematically changing as a function of composition in a way that at first glance, it is possible to interpolate between the *E_a_*-spectra with respect to *G′* and *δ* for each blend by using the pure blend components and a linear mixing rule.

This paper will analyze the rheological behavior of different combinations of blends of long-chain branched mPE, where three different low molar mass LCB-mHDPEs with different molar mass distributions are blended with a high molar mass LCB-mHDPE at different blending ratios. The paper focuses on understanding the thermorheological behavior caused by the interactions in such blends.

## 2. Experimental

### 2.1. Materials and Sample Preparation

The materials used for blending were synthesized on a laboratory scale by SABIC Limburg B.V. (Geelen, the Netherlands) using supported metallocene catalysts. All materials are polyethylene homopolymers (HDPE) to avoid any uncertainties concerning immiscibilities due to different comonomer contents. Table 1 lists the molecular data (*M_w_*, *M_z_*, *M_w_*/*M_n_*, and the LCB-content from SEC-MALLS) of the base materials, showing that they vary the molar mass *M_w_* and molar mass distribution *M_w_*/*M_n_* [49,50,51,52,53].

The solution method was chosen to avoid the known problem: it is challenging to properly melt-mix two polymers with significantly different viscosities [51].

To reach a total mass of polymer of 5 g, it was calculated how much HDPE (A–C) and HMW-HDPE (high molecular weight) needed to be mixed. The prepared dry blend was then added into a round bottom flask containing 85 mL p-xylene (GC grade, Macklin, Shanghai, China). The reflux condensation device was set up and the flask was placed in an oil bath at 120 °C for stirring. The polymers were gradually dissolved, turning the mixture transparent without any visible aggregates within 30 min. After stirring for 2 h, the blend solution was slowly poured into a beaker containing 1.5 L deionized water, which was continuously stirred at room temperature for 30 min to obtain HDPE polymer flocs. The pouring was performed so that only a thin stream or tiny drops of the solution were added to the stirred water to make the precipitation as fast as possible and avoid possible separation during precipitation. The formed precipitate consisted of fluffy particles with the largest dimension of ca. 2 mm after drying.

The solvent of p-xylene was removed twice by washing with ethanol in a Büchner funnel and then washed with deionized water 3 times. Following this, the blending particles were oven-dried *in vacuo* at 80 °C for 48 h; afterward, the milky white powder blending samples were obtained.

Table 2 contains the mixture names, which were systematically chosen according to the scheme A–C#, where A–C stands for the low molecular partner HDPE A–C and the #stands for the weight content of HMW-HDPE added; e.g., a mixture of 94% HDPE C and 6% HMW-HDPE is called C6.

The samples were pressed on a Beijing Future Material vacuum mold pressing machine (FM450, Beijing, China). The diameter and thickness of the shear rheological test samples are 25 mm and 1–1.5 mm, respectively.

### 2.2. Rheology

The rheological characterization was carried out on an Anton Paar MCR 702 rheometer (Graz, Austria) equipped with a forced convection oven. All shear rheological experiments were carried out in a nitrogen atmosphere.

The shear rheological experiments consisted of frequency sweeps in the range of *ω* = 100 … 0.1 rad/s and a deformation *γ*_0_ of maximum 5%, which is in the linear range of deformation for all samples. For HMW-HDPE, *γ*_0_ = 5% proved to be too high, owing to the very high viscosity and, thus, the deformation was lowered to 1%. These experiments were carried out at 150, 170, 190, 210, and 230 °C, and finally repeated at 150 °C to confirm that no thermal degradation had taken place. Before each experiment, the temperature was equilibrated within ±0.5 K of the desired temperature and followed by a waiting time of 300 s to ensure proper temperature equilibration in the sample.

The obtained data were analyzed in particular for their thermorheological complexity, which was done by the authors’ automated method previously described in ref. [43]. In this method, the rheological quantities selected storage modulus *G′*, loss modulus *G″*, and phase angle *δ*, are not shifted globally but slicewise to determine the Arrhenius activation energy *E_a_*, i.e., e.g., the activation energy *E_a_* is determined at *G′* = 10,000, 5000, and 2000 Pa [15,41]. Practically, a Matlab^®^ script is used to perform the following steps: 1. Fit log*G′*(log ω), log*G″*(logω), and *δ*(logω) with 4th or 5th order polynomials (5th order polynomials were only used in the rare cases when a 4th order polymer was insufficient for fitting of *δ*(logω)), 2. Find the intersection points of these polynomials with a list of constant values of log*G′*, log*G″*, and *δ*, 3. Determine the *E_a_* for each of those constant values.

## 3. Results

### 3.1. Shear Rheological Properties

#### 3.1.1. Base Materials

**Figure 1:**
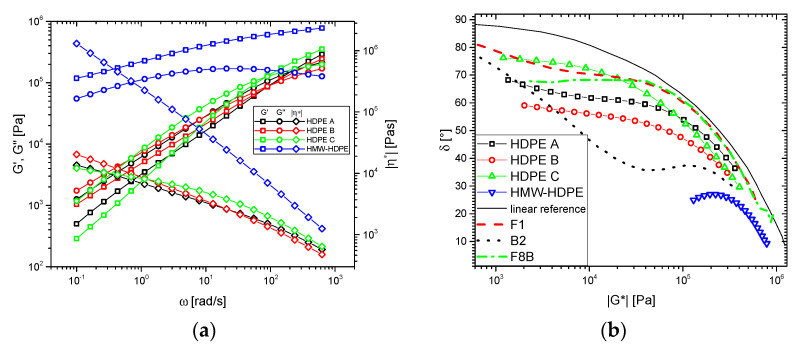
(**a**) Dynamic-mechanical modulus and viscosity functions of the individual blend components. (**b**) corresponding *δ* (|G*|)-plots. The linear reference (black line) was defined elsewhere for polyethylene homopolymers (HDPEs) and with *M_w_*/*M_n_* = 2–3 [57]. T = 150 °C, *γ*_0_ ≤ 5%. F1–*M_w_* = 102,000 g/mol, *M_w_*/*M_n_* = 2.0, low LCB-level, B2–*M_w_* = 93,000 g/mol, *M_w_*/*M_n_* = 1.9, high LCB-level, F8B–*M_w_* = 190,000 g/mol, *M_w_*/*M_n_* = 2.0, medium LCB-level, 1.8 mol% octene as comonomer [52]. The data at other temperatures are given in Supplementary Materials Figure SI1.

The rheological data of the blend components are given in Figure 1 and show that HDPE A, B, and C, although differing significantly in molar mass, are relatively similar in their rheological behavior, i.e., |η*|(*ω*), *G′*(*ω*), and *G″*(*ω*) are similar. The samples were chosen from a larger number of materials to have different *M_w_*, molar mass distributions and degrees of long-chain branching, while not showing a largely different rheological behavior. As shown in Table 1 and Figure 1, HDPE A is relatively low molecular (*M_w_* = 87,000 g/mol) and has a relatively high degree of long-chain branching, as becomes evident from the high crossover frequency *ω_c_* = 201 rad/s, which would be expected for a linear polymer with *M_w_* = 87,000 g/mol [7], i.e., the molar masses from GPC-MALLS and the rheological estimate are almost identical. Further, it has a non-negligible deviation from the linear reference in the *δ*(|G*|)-plot, which is equivalent to the parallelism of *G′*(*ω*) and *G″*(*ω*) and the almost constant slope of the magnitude of the complex viscosity function |η*|(*ω*) for *ω* < 10 rad/s. The relatively narrow molar mass distribution of HDPE A combined with the low *M_w_* = 87,000 g/mol leads to an upturn of *δ* in the *δ*(|G*|)-plot at relatively high |G*|, which is typical for relatively low molecular LCB-mPE [52]. For HDPE B with the somewhat higher *M_w_* = 105,000 g/mol, the shape of the *δ*(|G*|)-plot has changed significantly. Firstly, the plateau in *δ* is lower, but even more importantly, the upturn towards the lowest frequencies *ω* (→lowest |G*|) has almost completely disappeared. This becomes more evident when comparing the upturn with that of samples LCB-mHDPE F1 and B2, which were reported before to have almost the same *M_w_* (94,000 and 102,000 g/mol, respectively) but *M_w_*/*M_n_* = 2 [52,53], it becomes clear that that the broader molar mass distribution of HDPE A and in particular B leads to significantly broader transitions.

HDPE C is significantly higher in *M_w_* (180,000 g/mol) while having almost the same molar mass distribution *M_w_*/*M_n_* = 14.1 as HDPE B. The viscosity function |η*|(*ω*) for *ω* < 10 rad/s only has a relatively low slope in comparison, so that for the lowest frequencies, |η*|(*ω*) of HDPE C is lower than that of HDPE A and B with a higher degree of branching and a much lower molar mass. For comparison with a material with approximately the same *M_w_* but lower *M_w_*/*M_n_*_,_ the LCB-mLLDPE F8B was chosen (*M_w_* = 190,000 g/mol, *M_w_*/*M_n_* = 2, octene content 1.8 mol%) [54,55]. The expected value of zero shear-rate viscosity *η*_0_ for a linear polymer of equivalent molar mass *M_w_* to HDPE C (*M_w_* = 180,000 g/mol) is ca. 80,000 Pa s, which is significantly above the viscosity |η*|(*ω* = 0.1 rad/s) found for HDPE C. For sparsely long-chain branched polymers, in general, the zero shear-rate viscosity *η*_0_ is higher than those of their linear equivalent [56]. Consequently, the high molecular chains lead to a significant increase in the viscosity function |η*|(*ω*) at frequencies *ω* ≪ 0.1 rad/s, which is the smallest frequency *ω* used in this paper.

HMW-HDPE shows an approximately parallel *G′*(*ω*) and *G″*(*ω*) below *ω* = 1 rad/s and a *δ*(|G*|)-plot, where *δ* is decreasing with decreasing *ω*, i.e., *dδ*/*dlogω* > 0. Both are typical features of very high molecular LCB-PEs with low to medium long-chain branching [53]. Owing to the high molar mass *M_w_* and the relatively small temperature dependence of PE, it is impossible to reach the terminal regime for such materials within a reasonable time [52].

**Figure 2:**
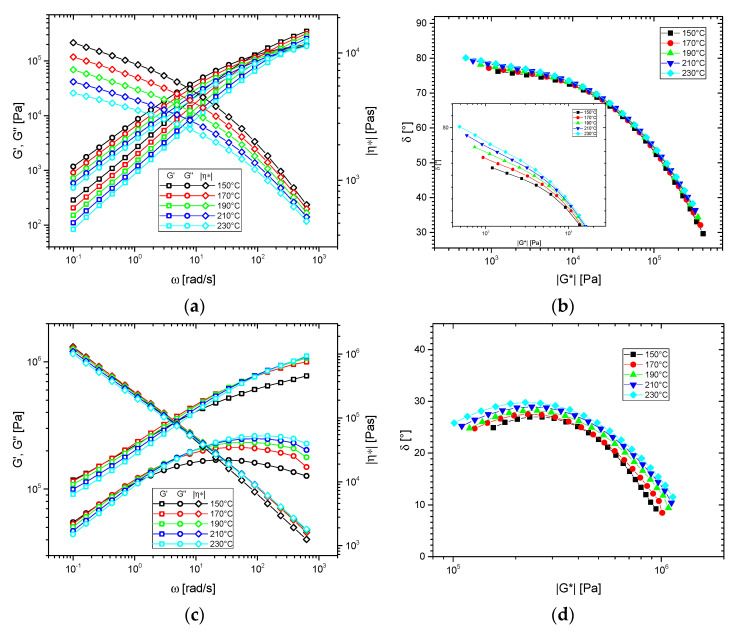
(**a**) dynamic-mechanical modulus and viscosity functions of HDPE C. (**b**) corresponding *δ*(|G*|)-plots. (**c**) *G′*(*ω*), *G″*(*ω*), and |η*|(*ω*) for HMW-HDPE and (**d**) corresponding *δ*(|G*|)-plots. *γ*_0_ ≤ 5%.

The thermorheological analysis for these samples was conducted following Stadler et al.’s method [43], which will be demonstrated in the following. The temperature-dependent dynamic-mechanical data for HDPE C (Figure 2) demonstrate that-like usually found for LCB-mPEs-the *G′*(*ω*), *G″*(*ω*), and |η*|(*ω*) functions appear to be shiftable at first glance to create a master curve. However, the *δ*(|G*|)-plot (Figure 2b) shows clearly that the data are indeed thermorheologically complex, as in the region plateau, characteristic of LCB-mPE, the *δ*(|G*|)-functions are temperature-dependent with the phase angle *δ* exhibiting lower values at the same complex modulus magnitude |G*|. This behavior of the sample, albeit more broad in molar mass distribution, corresponds to a **BL**-material (LCB-mPE) typical behavior according to the thermorheological complexity classification scheme [58]. Thermorheological complexity of type **BL** is characterized by curve shapes on the *δ*(|G*|)-plots similar to the shapes shown in Figure 1b, whose data in the plateau/shoulder region is highly temperature dependent. For those materials, the activation energy spectrum with respect to the phase angle *E_a_*(*δ*) typically show a very prominent peak, as will be shown later (Figure 2). HDPE A and B qualitatively show the same characteristics (Figure SI1).

The thermorheological complexity of very high molecular metallocene materials (above *M_w_* = 250,000 g/mol) has not been reported so far to the best of our knowledge. HMW-HDPE shows an almost temperature-independent viscosity function (Figure 2c), while the modulus functions show a temperature dependence, which can be explained with the smaller dynamic range and the temperature and frequency dependence of the phase angle. The *δ*(|G*|)-plots show that the higher the temperature, the higher is the phase angle *δ* of the peak around 27°, which occurs before the minimum in the *δ*(|G*|)-plot, which is outside the measurement range, but has to be present in the measurement data, according to experiences with similar materials [52,53,55]. In the case of HMW-HDPE, this minimum and the following upturn towards *δ* = 90° is located at too small frequencies to be measured within a reasonable time (>1 day). Taking this somewhat different curve shape into account, it can be concluded that otherwise, HMW-HDPE thermorheologically behaves just like a BL-material (LCB-mPE) according to the thermorheological complexity classification scheme [58].

So, in conclusion, HDPE A, B, and C, as well as HMW-HDPE, behave like typical LCB-mPE, albeit with somewhat more smeared out curves, owing to their broader molar mass distribution than previously published data on LCB-mPE.

The thermorheological complexity analysis will be discussed in detail, together with the blends made from those base materials to avoid repetition.

#### 3.1.2. Blends

**Figure 3:**
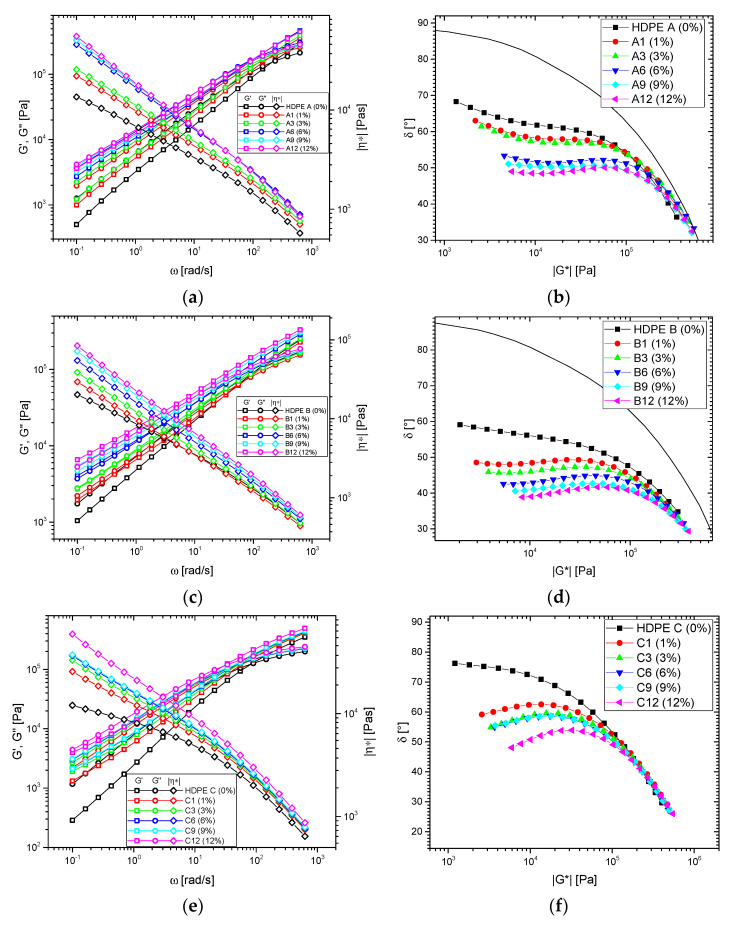
(**a**) dynamic-mechanical modulus and viscosity functions of the blends of (**a**) HDPE A, (**c**) HDPE B, and (**e**) HDPE C. Corresponding *δ*(|G*|)-plots for (**b**) HDPE A, (**d**) HDPE B, and (**f**) HDPE C. T = 150 °C, *γ*_0_ = < 5%.

The frequency-dependent data of the three base materials and 15 blends are given in Figure 3. In all cases, the addition of the high molecular HMW-HDPE leads to distinct changes in rheological properties. Even for 1 wt.% HMW-HDPE-content, a clear increase of the viscosity and moduli functions are found at low frequency, which, as expected, is where a high molecular component has the strongest influence.

The HDPE A blends roughly retain the typical shapes of a narrow molar mass distribution LCB-mPE (Figure 3a,b, Figure SI2 (T = 170–230 °C)). Their behavior is characterized by a viscosity function |η*|(*ω*) with more or less constant slope, which then levels of towards the zero shear-rate viscosity *η*_0_ [59,60]. However, this leveling off was not found for any sample in this paper, as the focus was on thermorheological behavior. In the *δ*(|G*|)-plot, the addition of HMW-HDPE leads to a decrease of the *δ*-plateau and further flattening of its slope due to the introduction of long relaxation modes by the blending. These effects are equivalent to each other, as through Kramers-Kronig relations *δ*(*ω*) and dlog|η*|/dlog(*ω*) are inversely proportionally related [59,60,61,62]. For the highest HMW-HDPE contents, the *δ*(|G*|)-plots start exhibiting a small minimum.

HDPE B and its blends do not show the leveling mentioned above to *η*_0_ and a clear end of the *δ*-plateau (Figure 3c,d, Figure SI3 (T = 170–230 °C)) due to its broader molar mass distribution. This leads to almost perfectly straight viscosity functions. Consequently, it is nearly impossible to estimate the zero shear-rate viscosity *η*_0_ of these blends.

HDPE C has a lower long-chain branching efficacy than HDPE B, but a similar broad molar mass distribution. As a consequence, the viscosity functions are not as straight as for the HDPE B series. However, due to the higher molar mass of HDPE C compared to HDPE B, the terminal relaxation time leads to even broader transitions in the *δ*(|G*|)-plot than the HDPE B blend series. The HDPE C-blend series (except pure HDPE C) does not show a single material whose *δ*(*ω*) has a negative slope at low *ω*, i.e., all materials have a maximum at higher frequencies and a minimum at *ω* < 0.1 rad/s (Figure 3e,f, Figure SI4 (T = 170–230 °C)).

**Figure 4:**
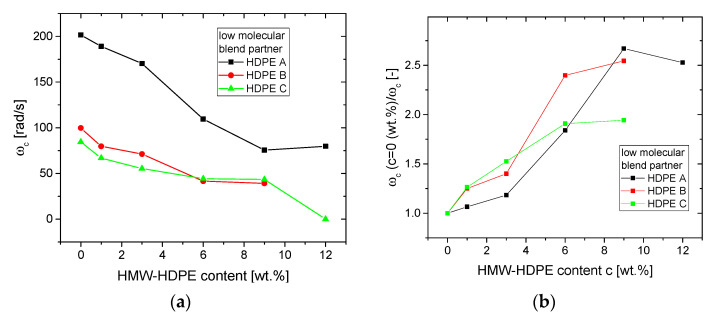
(**a**) Crossover frequencies *ω_c_* for the blends as a function of HMW-HDPE content *c*, (**b**) ratio of *ω_c_* of pure low molecular blend partner and the respective blends.

The crossover frequencies *ω_c_* for the blends (Figure 4) show a clear decrease with increasing HMW-content *c*. Considering that *ω_c_* is proven to be related to *M_w_* [7,63], it is not surprising that blending HMW-HDPE will increase *M_w_* and, thus, also decrease *ω_c_*. However, these correlations between *ω_c_* (or inverse characteristic relaxation time from the Carreau-Yasuda model 1/λ ≈ *ω_c_* [64,65]) and *M_w_* are influenced by various factors, most notably by the molar mass distribution. The molar mass distribution changes when blending two materials. However, it is clear that *ω_c_*(*c*) (Figure 4a) shows a common trend for all blend series. It should be noted that those correlations between *ω_c_* (or 1/λ) and *M_w_* have been established for linear and not for long-chain branched PE. However, as long as the deviation from the linear relation in the *δ*(|G*|)-plot (as shown in Figure 3) is not significant for *δ* < 50 °C (i.e., at *ω* = *ω_c_* (*δ* = 45°), the rheological data are not influenced by the long-chain branches), it is safe to relate *ω_c_* with *M_w_*, which is in line with fits of the viscosity functions of narrow molar mass distribution LCB-mPEs [66]. This becomes clear for the sample C12, whose *δ*(*ω*)-function exhibits a peak around 52° before decreasing again with decreasing *ω* (Figure 6). B12 has a very similar behavior but does not exceed 45°, thus does not have a crossover frequency *ω_c_*.

When calculating the ratio of *ω_c_* of pure low molecular blend partner and the respective blends, it becomes clear that, although somewhat scattered, the slope of the decrease function is independent of the base material, which is surprising, considering that the molar mass *M_w_* ratios of the blends’ base materials are between 5.4 for the HDPE A-HMW-HDPE and 2.6 for the HDPE C-HMW-HDPE blend series.

Assuming that the materials are monodisperse and that the crossover frequency *ω_c_* is only related to *M_w_*, it would be possible to calculate the expected *M_w_* and thus *ω_c_* (~*M_w_*^−3.6^) simply from the constituents’ *M_w_* and contents. Consequently, the HDPE A series *ω_c_* should decrease to *ω_c_*/*ω_c_*(*c* = 0 wt.%) ≈ 0.21 and for HDPE C to ≈ 0.40 for *c* = 12 wt.%. However, such a big difference is not found for the blends, which indicates that the influence of molar mass distribution on blends needs a lot more understanding that cannot be broken down to such a primitive approach.

**Figure 5:**
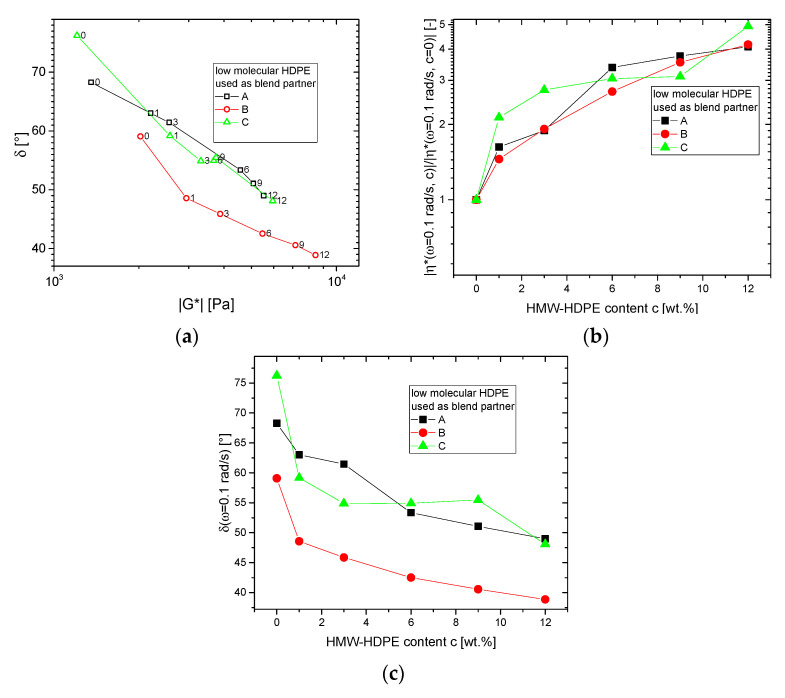
Comparison of the dynamic-mechanical data obtained for the base materials and blends at *ω* = 0.1 rad/s and T = 150 °C. The concentration of HMW-HDPE *c* is denoted as a number next to each datapoint. (**a**) *δ* (|G*|)-plot, (**b**) viscosity change normalized to viscosity of low molecular blend partner |η*(*ω* = 0.1 rad/s, (**c**) |/|η*(*ω* = 0.1 rad/s, *c* = 0)|, (**c**) *δ*(*ω* = 0.1 rad/s).

The differences induced by adding HMW-HDPE to the low molecular HDPEs become much more pronounced when looking at the lowest frequencies, however (Figure 5). The *δ*(|G*|)-plots only for *ω* = 0.1 rad/s show a clearly decreasing trend for the phase angle *δ*, while the complex modulus |G*| increases monotonously as a function of HMW-HDPE content *c* (Figure 5a). The complex viscosity |η*| at *ω* = 0.1 rad/s as a function of HMW-HDPE content normalized to the low molecular blend partners’ |η*|, shows an increase by about factor 4 for the HDPE A–C blends (Figure 5b).

Figure 5c shows the dependence of phase angle *δ* on HMW-HDPE content *c*, which for larger *c* leads to an almost parallel dependence, while for small *c*, the initial decrease of *δ* is very much sample dependent.

This indicates that at the low frequencies, even a tiny amount of HMW-HDPE in the blends has a significant effect on the viscoelastic behavior, which is qualitatively in line with the strong increase of the elastic compliance found by Münstedt [67]. Further, it indicates that the larger the molar mass difference is, the larger is the effect on the rheological properties, especially at low frequency.

### 3.2. Thermorheological Behavior

**Figure 6:**
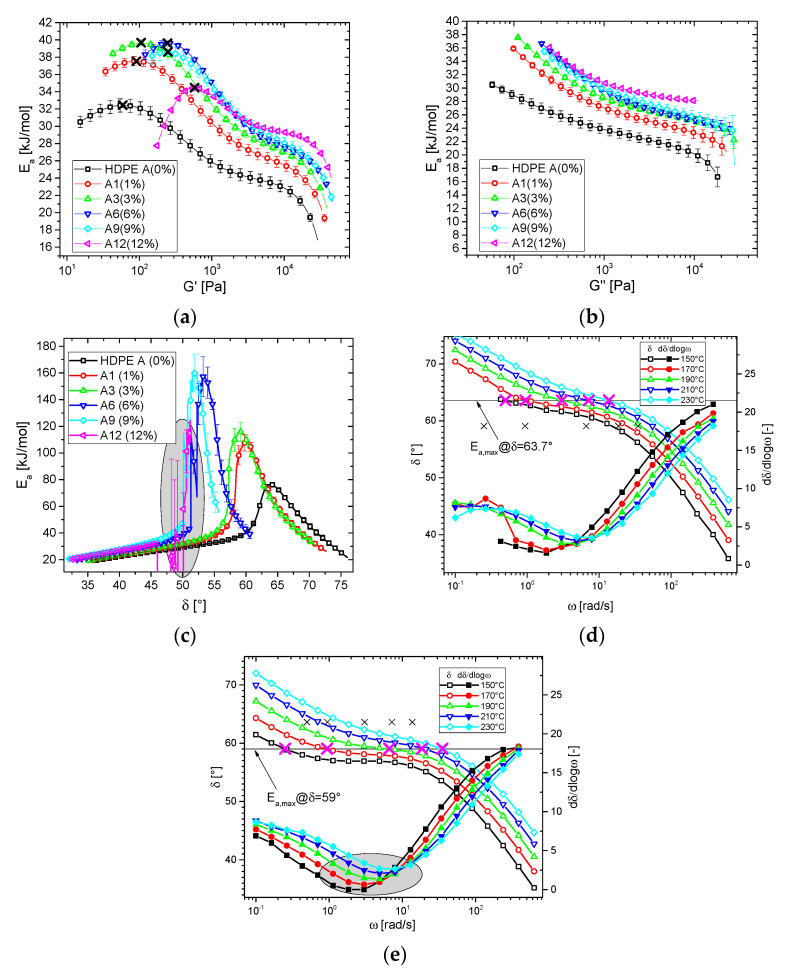
Activation energy spectra of (**a**) storage modulus (*E_a_*(*G′*)), (**b**) loss modulus (*E_a_*(*G″*)), and (**c**) phase angle (*E_a_*(*δ*)). (**d**) *δ*(*ω*) of HDPE A, (**e**) *δ*(*ω*) of A3. The pink × in (**d**) and (**e**) denote the points at which the *δ*(|G*|)-plots reach the phase angles *δ*, at which *E_a_*(*δ*) HDPE A and A3 have their peak values respectively. The black × in (**d**) and (**e**) correspond to the pink × in (**e**) and (**d**), respectively.

The analysis method for the thermorheological behavior was described in detail before [47] and will, therefore, not be repeated here. The results of the numerical analysis of the thermorheological complexity are given in Figure 6a–c. *E_a_*(*G′*) (Figure 6a) shows that blending HDPE A with HMW-HDPE leads to an overall increase of *E_a_* up to 6% HMW-HDPE-content *c*. Above this threshold, the activation energies above 30 kJ/mol are decreasing. For all samples, the data exhibits a maximum in *E_a_*(*G′*), whose *G’*-position increases with increasing HMW-HDPE-content *c*, i.e., the influence of the long-chain branches becomes visible at higher *G′*, which also corresponds to higher *ω*. This statement is true in general, as long-chain branches lead an increased elasticity, which increases the creep recovery compliance [67,68] and *G′* at low *ω* [50,69,70,71] owing to the introduction of long relaxation modes into the relaxation spectrum [72].

The loss modulus activation energy spectrum *E_a_*(*G”*) shows an increase of *E_a_* towards decreasing *G″*, which is due to the increasing efficacy of long-chain branches towards low frequencies (caused by long relaxation times due to LCBs) [42,72]. Increasing the blend concentration *c* leads to an overall increase of *E_a_*(*G″*) while mostly retaining the shape of the loss modulus activation energy spectrum.

Like in previous reports [15,22,43,58], it was also found that *E_a_*(*G′*) and *E_a_*(*G″*) is “too low” for high *G′* and *G″*, i.e., the observed values of *E_a_* are significantly below the expected value of *E_a_* for linear HDPE (27 kJ/mol). In previous papers, such low *E_a_* were also found for the relaxation strength activation energy spectrum *E_a_*(H) [31,41]. This effect is not found for linear materials when evaluating thermorheological complexity with the slice-wise method; thus, it is most likely not any form of experimental artifact. However, so far, nobody has presented a conclusive mechanism for observing such “too low” *E_a_*_,_ and a credible explanation for this effect has not been found, which is why regrettably this question has to remain unexplained.

The activation energy spectra with respect to *δ E_a_*(*δ*) shows a clear peak function (Figure 6c), whose peak heights go through a maximum when increasing the HMW-HDPE-content *c*, while *δ*_max_, which has been proven to be closely related to the characteristic phase angle *δ*_c_, according to the definition of Trinkle et al. [73,74], decreases with increasing *c*. Those effects are expected, as adding HMW-HDPE leads to an increase in long relaxation times, which–in general–broadens the relaxation spectrum and, thus, leads to increased elasticity, i.e., a lower plateau in the *δ*(|G*|)-plot or *δ*(*ω*)-plot. However, unlike for “normal LCB-mPE”, a decrease in *δ*_max_ is usually accompanied by a moderate increase in the peak activation energy of *E_a_*(*δ*) of *E_a_,_max_*. The values *E_a_,_max_* and *δ*_c_ found for HDPE A are 91.7 kJ/mol and 63.7°, respectively, which is in good agreement to previously reported values for similar materials [43]. However, the blends show much higher values of *E_a_,_max_* than expected, which is counterintuitive at first glance, as adding an HMW-HDPE broadens the molar mass distribution, which according to previously reported results, “smears out the plateaus” in the *δ*(|G*|)-plot or *δ*(*ω*)-plot and thus should rather decrease than increase *E_a_,_max_* [43]. In other words, the previous results for LCB-mPEs with a unimodal broad molar mass distribution were exactly the opposite of the results found in this paper with respect to *E_a_*(*δ*).

It should be mentioned that due to the piecewise evaluation, the activation energies are highly influenced by their input. In the plateau regime of *δ*(*ω*), the values of *E_a_*(*δ*) can increase significantly beyond the values of *E_a_*(*G′*) and *E_a_*(*G*″**). This peak in *E_a_*(*δ*) is, of course, not supposed to be taken as a physical value but rather as an indication that is very valuable for branching analysis.

Figure 6d and e show a comparison of the *δ*(*ω*)-plots of HDPE A and A3. In these plots, the position of *E_a_,_max_* (*δ*_max_ = 63.7° and 59°, respectively) is marked by the horizontal line, and the pink x marks the points where *δ*(|G*|; T = 150, 170, 190, 210, and 230 °C) cross this value. Further, the other material’s corresponding values are given in the respective plots as small black × to illustrate the differences better. The comparison between these two samples, differing by the 3% addition of HMW-HDPE, leads to significant differences in the phase angle functions *δ*(*ω*). Namely, the plateau of A3 is significantly widened and flattened to the extent that for 150 °C, an almost constant value of *δ* is found for *ω* = 1 … 10 rad/s. As a consequence, the spread between the data at 150 °C and 230 °C at *δ* = 59° is significantly larger than the corresponding spread for HDPE A at *δ* = 63°.

Thus, the high molecular tail introduced by blending leads to significantly different behavior than found for the “normal” broadly distributed LCB-mPE. The high molecular tail stemming from 1–12% HMW-HDPE, which in the case of HDPE A is 5.4 times higher with respect to *M_w_*, leads to a significant lengthening of the terminal relaxation time and to an increase in perceived LCB-efficacy.

For HMW-HDPE contents higher than 3%, some erratic peak functions were found, resulting from *δ*(*ω*) exhibiting a minimum, as can be seen from Figure 3a,b. As the evaluation routine finds one frequency, at which the phase angle *δ* is exactly at a defined phase angle, the analysis is not adequately defined, as the slice-wise evaluation assumes that all evaluated functions are monotonically increasing or decreasing. For this reason, the values found for samples having a minimum in *δ*(*ω*) cannot be trusted in the regime of the minimum, which is indicated by the gray ellipse in Figure 6c.

Despite these limitations owing to the slice-wise evaluation approach, it is possible to determine *E_a_,_max_* for A6 and A9 but not for A12, whose *δ*-minimum is too pronounced to yield reliable values. *E_a_,_max_* of A6 and A9 are around 160 kJ/mol, which is beyond any *E_a_* reported for thermorheologically complex polyethylene. This illustrates the strong effect of the broadening of the spectrum by blending, which leads to bimodal materials. Figure 6e clearly shows that *δ*(*ω*) of A3 at 150 °C has a plateau (*dδ*/*dlogω* = 0), while at 230 °C, the smallest slope is around 2.6, as can be seen from the phase angle derivative plots (*dδ*/*dlogω*(*ω*), Figure 6d,e). For HDPE A (Figure 6d) the corresponding minima are 1.6 and 3.5, respectively.

For these reasons, the thermorheological complexity of the other blend series is only discussed in terms of *E_a_*(*G′*) and *E_a_*(*δ*). The plots of the other quantities are given in supplementary information.

**Figure 7:**
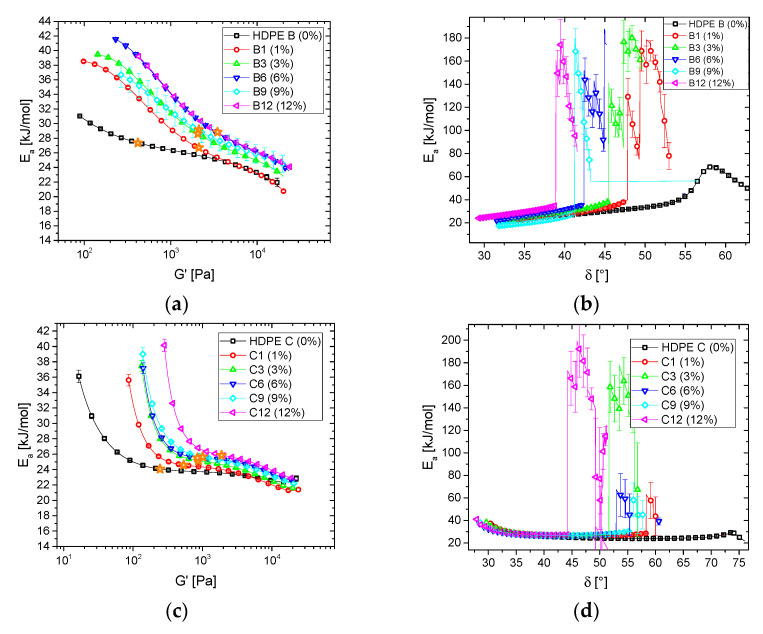
Activation energy spectra of (**a**,**c**) storage modulus (*E_a_*(*G′*)), (**b**,**d**) phase angle (*E_a_*(*δ*)) of (**a**,**b**) HDPE B blend series, (**c**,**d**) HDPE C blend series.

Figure 7 shows *E_a_*(*G′*) and *E_a_*(*δ*) of the blend series B–D. The HDPE B blend series shows the largest similarity to the HDPE A blend series (Figure 7a,b). Compared to the HDPE A blend series, the higher molar mass and longer terminal relaxation time of the HDPE B blend series increase the storage modulus activation energy spectrum *E_a_*(*G′*) for low *G′*, while the influence at high *G′* is rather low. This and the higher branching efficacy of HDPE B vs. HDPE A make the samples approach higher *E_a_*-values as seen from the higher slopes found when approaching the maximum. Further, the deviation from the *E_a_* of linear materials (ca. *E_a_* = 27 kJ/mol) occurs at somewhat higher *G’*. *E_a_*(*δ*) clearly shows that the typical peak shifts towards lower *δ* with increasing *c*. However, only for the pure HDPE B could a regular *E_a_*(*δ*) peak could be found. A minimum in *δ*(*ω*) exists for all blends, which, as discussed before, leads to extremely high *E_a_,_max_* and erratic peak shapes. Thus, the obtained values for, e.g., *E_a,max_* cannot be considered quantitative, but it can be concluded that they are very high and that *δ*_max_ decreases with increasing *c*. The orange star symbols in Figure 7a are the inflection points, which will be discussed in Figure 9.

**Figure 8:**
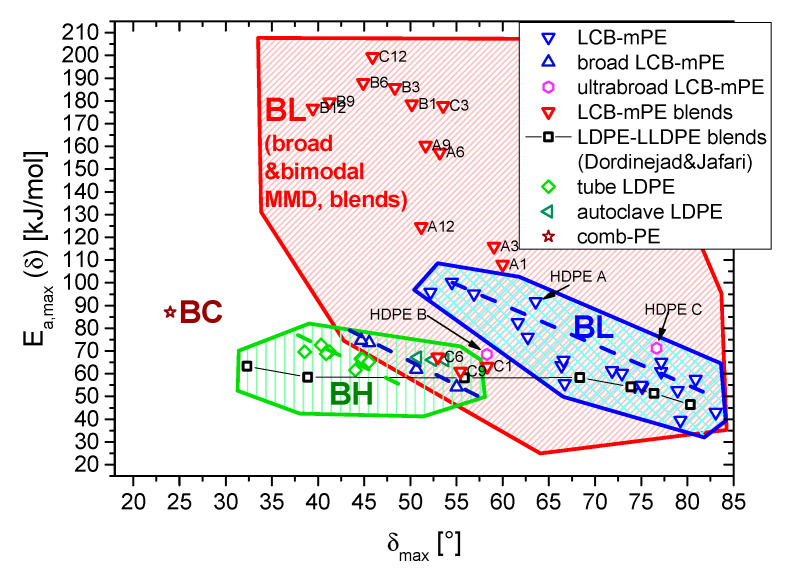
Plot of *E_a_,_max_* vs. *δ*_max_ for the samples in comparison with previously characterized samples. The samples included in this paper are denoted with their sample designation. The samples not designated in particular are literature data [18,43,58].

Figure 8 shows the *E_a_,_max_*(*δ*_max_) plot for all samples investigated by the authors and some literature values. The topographies (1) branched with few long-chain branches (LCB-mPE)→BL, (2) highly long-chain branched (LDPE)→BH, and branched with comb-like topography→BC are marked clearly as areas in the same color as the respective symbols for individual samples [58].

Previously, it was established that LCB-mPE with narrow molar mass distribution (BL, blue downward-pointing triangles) have a significantly higher *E_a_,_max_*(*δ*_max_) than LCB-mPE with a broader molar mass distribution (dark blue upward-pointing triangles), whose *E_a_,_max_*(*δ*_max_) is rather close to tubular and especially autoclave LDPE [43]. It was then possible to accumulate some more data, particularly for the LDPE-LLDPE blends of Dordinejad and Jafari [18], which were analyzed with the same standard analysis method. This data set shows a transition from LDPE-like behavior (BH) for the LDPE-rich blends to LCB-mPE like behavior (BL). The LLDPE used was a Ziegler-Natta grade with broader molar mass distribution, but according to the data published (*G′*(*ω*), *G″*(*ω*), *E_a_*(*G’*), *E_a_*(*δ*), …) [25] slightly long-chain branched, making it sufficiently similar to a slightly branched LCB-mPE.

When comparing the previously obtained data to the LCB-mPE blends, it becomes immediately obvious that the special curve shape of *δ*(*ω*) leads to *E_a_,_max_*(*δ*_max_) very different from previously known samples, owing to their very high *E_a_*(*δ*)-peaks. This confirms the unusual behavior of the LCB-mPE blends, which is due to the bimodal molar mass distribution with long-chain branches in the high molecular tail. Thus, the blue BL-area has to be considered to be a special case of a much broader range of for lightly branched materials with non-standard molar mass distributions (i.e., symmetrical molar mass distributions with *M_w_*/*M_n_* = 2…4). The blends of the HDPE A and HDPE B-series exhibit a somewhat lower *δ*_max_ and higher *E_a_,_max_* than normal BL-materials, while the HDPE C-blend series is rather similar to the broad molar mass distribution LCB-mPEs and autoclave LDPEs published before [43]. This difference is probably due to the broad molar mass distribution of HDPE C and the relatively small *M_w_* difference between HDPE C and HMW-HDPE.

Those new data clearly indicate that the previously relatively narrow range typically found for BL-materials (blue area) has to be significantly expanded to form a BL-category comprising also broad and bimodal molar mass distributions (including blends), which is marked as the red area in Figure 8.

**Figure 9:**
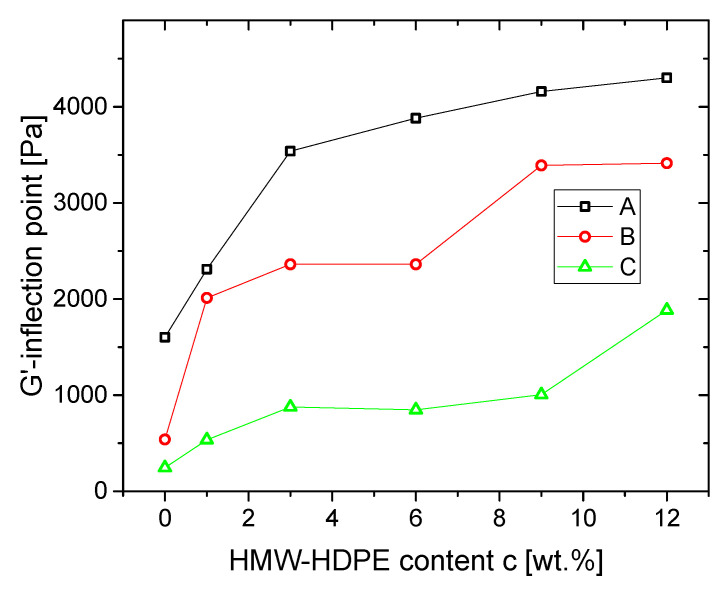
*E_a_*(*G′*)-inflection point plotted as a function of HMW-HDPE content *c* [wt.%].

Besides the discussion of *E_a_*(*δ*), also *E_a_*(*G′*) can offer interesting insights into the rheological behavior. When looking at Figure 6a and Figure 7a,c carefully, the point at which the samples have a clear upturn in *E_a_*(*G′*) depends on the low molar mass blend partner and blend composition (marked in Figure 7a as orange stars). To analyze this behavior better, the turning points were determined for each sample and the *G′* component of the inflection point is given in Figure 9.

Two trends are immediately visible: firstly, an increase of HMW-HDPE content leads to an increased inflection point, i.e., the activation energy *E_a_* increases at a higher storage modulus beyond the level of linear material. Thus, the influence of the long-chain branches appears at higher *G′* and, consequently, at higher *ω*. This effect is exceptionally well visible at low HMW-HDPE contents. This is in agreement with observations of Münstedt [67] of minute amounts of a high molecular component having a massive influence on the elasticity of a blend, for which *G′* is the indicator used in this paper.

Secondly, the higher the molar mass of the low molecular material, the lower the *G′* at which the inflection point curves are found. This trend can be understood only to be the consequence of the combination of several parameters. Firstly, as the HMW-HDPE is the same for all materials, a higher molar mass of the low molecular HDPE means that both molar mass ratio of the blend partners varies as HDPE A-blends > HDPE B-blends > HDPE C-blends. The difference between the low molecular base polymers HDPE A, HDPE B, and HDPE C is primarily in *M_w_*, *M_w_*/*M_n_*, and the LCB-content, which balances out the differences in molar mass, as the viscosity functions of the three base materials are very similar (Figure 1). The lower LCB-content of HDPE B and HDPE C leads to an inflection point at rather low *G′*. This indicates that the long-chain branches only become evident at low *G′*/low *ω*, indicating that they only have few, but long long-chain branches mostly stemming from HMW-HDPE, as can be expected from the high molar mass of HMW-HDP*E*.

## 4. Conclusions

Blends of two LCB-mPEs with different molar masses and molar mass distributions and long-chain branching were created by solution blending. The rheological analysis revealed that the behavior of the blends changes already significantly when adding a minute amount of high molar mass material.

A high molecular HDPE lowers the phase angle at low frequency systematically, which suggests an increased elasticity and an extended terminal relaxation time. The samples’ thermorheological complexity varied systematically with the content of HMW-HDPE through increased activation energies at low frequencies, i.e., at low *G′* and especially in the *δ*-peaks. Due to the change of the curve shape of *δ*(*ω*), the shape of the phase angle activation energy spectrum *E_a_*(*δ*) changed significantly as well. The long relaxation times introduced by HMW-HDPE led to a clear flattening or even the appearance of a minimum in *δ*(*ω*) at intermediate *δ* (30–60°, depending on the material), which leads to very high *E_a_*.

This is very different from the previously observed behavior for LCB-mPE with *M_w_*/*M_n_* ≈ 2–3 and broad molar mass distribution LCB-mPE with *M_w_*/*M_n_* ≈ 4–6, whose *E_a_,_max_* is lowered by the smearing out due to the broadening of the molar mass distribution. For those previously established materials, *E_a_,_max_* was found to be between 50 and 100 kJ/mol, depending on the maximum activation energy phase angle *δ*_max_ (approximately the same as the characteristic phase angle *δ*_c_) and an increase in *M_w_*/*M_n_* led to a significant decrease of *E_a_,_max_* for those broad LCB-mPEs, whose molar mass distribution is unimodal. For the bimodal samples characterized in this paper, *E_a_,_max_* is much higher, despite the much broader molar mass distribution, which–as stated before–should lower *E_a_,_max_* at first thought. The increase of *E_a_,_max_* is the consequence of the bimodal molar mass distribution introducing very long relaxation times, leading to *E_a_,_max_* up to 200 kJ/mol.

Therefore, it is concluded that the molar mass distribution *M_w_*/*M_n_* alone (of course together with the LCB-content and distribution) does not determine the thermorheologically complex behavior but that the shape of the molar mass distribution matters as well as its combination with the LCB-distribution, which together determine the relaxation spectrum and, thus, the viscoelastic functions.

The difference between broad molar mass distribution LCB-mPE and the LCB-mPE blends studied in this paper is clearly that the former have a very similar molar mass distribution and LCB-distribution to regular LCB-mPE (*M_w_*/*M_n_* ≈ 2), while the blends consist of 2 separately synthesized materials with their own molar mass distribution and LCB-topography. The high molar mass of HMW-HDPE also leads to very long long-chain branches, as the LCB-length in sparsely branched LCB-mPE approximately equals *M_n_* [75] and according to reports of star-branched materials, the arm-length (=LCB-length) has a much stronger effect on the terminal relaxation time than the weight average molar mass *M_w_* has [76]. Consequently, the introduction of HMW-HDPE leads to a much stronger lengthening of the terminal relaxation than expected from the molar mass difference alone. These very long terminal relaxation modes lead to an approximately constant slope in *G′*(*ω*) and *G″*(*ω*), which corresponds to a plateau (or small maximum, followed by a shallow minimum) in *δ*(*ω*), over a broad frequency range. As an increase in temperature leads to a systematic increase in this *δ*-plateau as well, very large shift factors are observed in the region of this plateau, which lead to the obsevered very high *E_a_,_max_*. The fact that typically the *δ*-plateaus are not 100% flat but show weak minima and maxima is also the reason for the erratic curve shapes of *E_a_*(*δ*); the numerical analysis finds one out of several intersection points of *δ*(*ω*) with a certain value of *δ* and for some values of *δ* for most of the blends there is more than one such intersection.

Broad molar mass distribution LCB-mPE do not have these very long terminal relaxation times but rather smeared out rheological data, which means that they do not have a spectrum that leads to a true *δ*-plateau but rather to a broad zone with constant but non-negligible slope and consequently a much lower *E_a_,_max_* than the blends and LCB-mPE with narrow molar mass distribution.

## Figures and Tables

**Table 1:** Molecular data of the base materials.

Material	*M_w_* [g/mol]	*M_z_* [g/mol]	*M_w_*/*M_n_* [−]	LCB from SEC-MALLS *
HDPE A	87,000	320,000	4.2	medium
HDPE B	105,000	1,400,000	15.8	medium-strong
HDPE C	180,000	5,400,000	14.1	none
HMW-HDPE 1	460,000	930,000	3.6	weak

* The LCB-content from SEC-MALLS is given qualitatively, only, due to the uncertainties in the Zimm-Stockmayer relation [49] and the fact that the resulting branching degrees from the Zimm-Stockmayer relation [49] are not molar mass independent. Previous rheological investigations have shown that materials that appear linear in SEC-MALLS can have long-chain branches in rheology [54].

**Table 2:** Designation of the blending materials.

Weight Content of HMW-HDPE 1 Added	HDPE A	HDPE B	HDPE C
1%	A1	B1	C1
3%	A3	B3	C3
6%	A6	B6	C6
9%	A9	B9	C9
12%	A12	B12	C12

## Data Availability

The raw data can be extracted from the provided graphs in SI or is available from the authors upon request.

## Supplementary Materials

The following are available online at https://www.mdpi.com/2073-4360/13/3/328/s1.

## List of Abbreviations

**Rheological quantities**	
Storage modulus	*G′*
Loss modulus	*G″*
Phase angle	*δ*
Complex viscosity function	*|η*|(ω)*
zero shear-rate viscosity	*η_0_*
Magnitude of the complex modulus	*|G*|*
Angular frequency	*ω*
Relaxation strength	*H*
Relaxation time	*τ*
Hencky strain rate	$\dot{\epsilon }\;$
crossover frequency	*ω_c_ (ω of G′=G″)*
temperature dependence according to Williams-Landel-Ferry’s law (based on free volume and not on potential barrier)	*WLF-temperature dependence*
shift factor	*a_T_*
Arrhenius activation energy	*E_a_*
Activation energy spectrum determined from shifting *δ(ω)*	*E_a_(δ)*
Activation energy spectrum determined from shifting *G’(ω)*	*E_a_(G′)*
Activation energy spectrum determined from shifting *G”(ω)*	*E_a_(G″)*
Maximum activation energy, determined from *E_a_(δ)*	*E_a_,_max_*
Phase angle at which E_a_,_max_ is found	*δ_max_*
Critical phase angle according to definition of Trinkle et al. [73]	*δ_c_*
Slope of phase angle	*dδ/dlogω*
Material with an LCB-mPE-like thermorheologically complex behavior [62]	LCB-mPE-like behavior (BL), BL-material
Material with an LDPE-like thermorheologically complex behavior [62]	LDPE-like behavior (BH), BH-material
Material with a comb-PE-like thermorheologically complex behavior [62]	comb-PE -like behavior (BC), BC-material
**Molecular quantities**	
number-average molar mass	*M_n_*
weight-average molar mass	*M_w_*
z-average molar mass	*M_z_*
Polydispersity index	*M_w_/M_n_*
molar mass distribution	*MMD*
long-chain branches	*LCBs*
**Material types**	
long-chain branched metallocene catalyzed polyethylene	*LCB-mPE*
long-chain branched metallocene catalyzed high densitypolyethylene	*LCB-mHDPE*
long-chain branched metallocene catalyzed linear low densitypolyethylene	*LCB-mLLDPE*
low-density polyethylene	*LDPE*
linear low density Ziegler-Natta polyethylene	*ZN-LLDPE*
high density Ziegler-Natta polyethylene	*ZN-HDPE*
**Miscellaneous**	
Logarithm (base 10)	*log*
Weight %	*wt.%*
