# Supplementary material for: Rheological Behavior of Blends of Metallocene Catalyzed Long-Chain Branched Polyethylenes. Part I: Shear Rheological and Thermorheological Behavior
Source: Polymers (Basel). 2021 Jan 20;13(3):328. doi: 10.3390/polym13030328 (PMC7864184; doi:10.3390/polym13030328)
Supplement: Supplementary file 1 [file polymers-13-00328-s001.pdf]

polymers

MDPI

Supplementary information

Rheological Behavior of Blends of Metallocene Catalyzed Long-Chain Branched Polyethylenes. Part I: Shear Rheological and Thermorheological Behavior

Chuangbi Chen, Mehdihasan I. Shekh, Shuming Cui and Florian J. Stadler*

College of Materials Science and Engineering, Shenzhen Key Laboratory of Polymer Science and Technology, Guangdong Research Center for Interfacial Engineering of Functional Materials, Nanshan District Key Laboratory for Biopolymers and Safety Evaluation, Shenzhen University, Shenzhen, 518055, China; 1810342023@email.szu.edu.cn (C.B.C.), mehdi.shekh3@yahoo.com (M.I.S.), 1910342023@email.szu.edu.cn (S.M.C.).

* Correspondence: fjstadler@szu.edu.cn

ORCID: CCB-0000-0001-9545-8767, MHS - 0000-0002-8214-6665, SMC-0000-0002-0514-368X, FJS-0000-0002-5849-1485

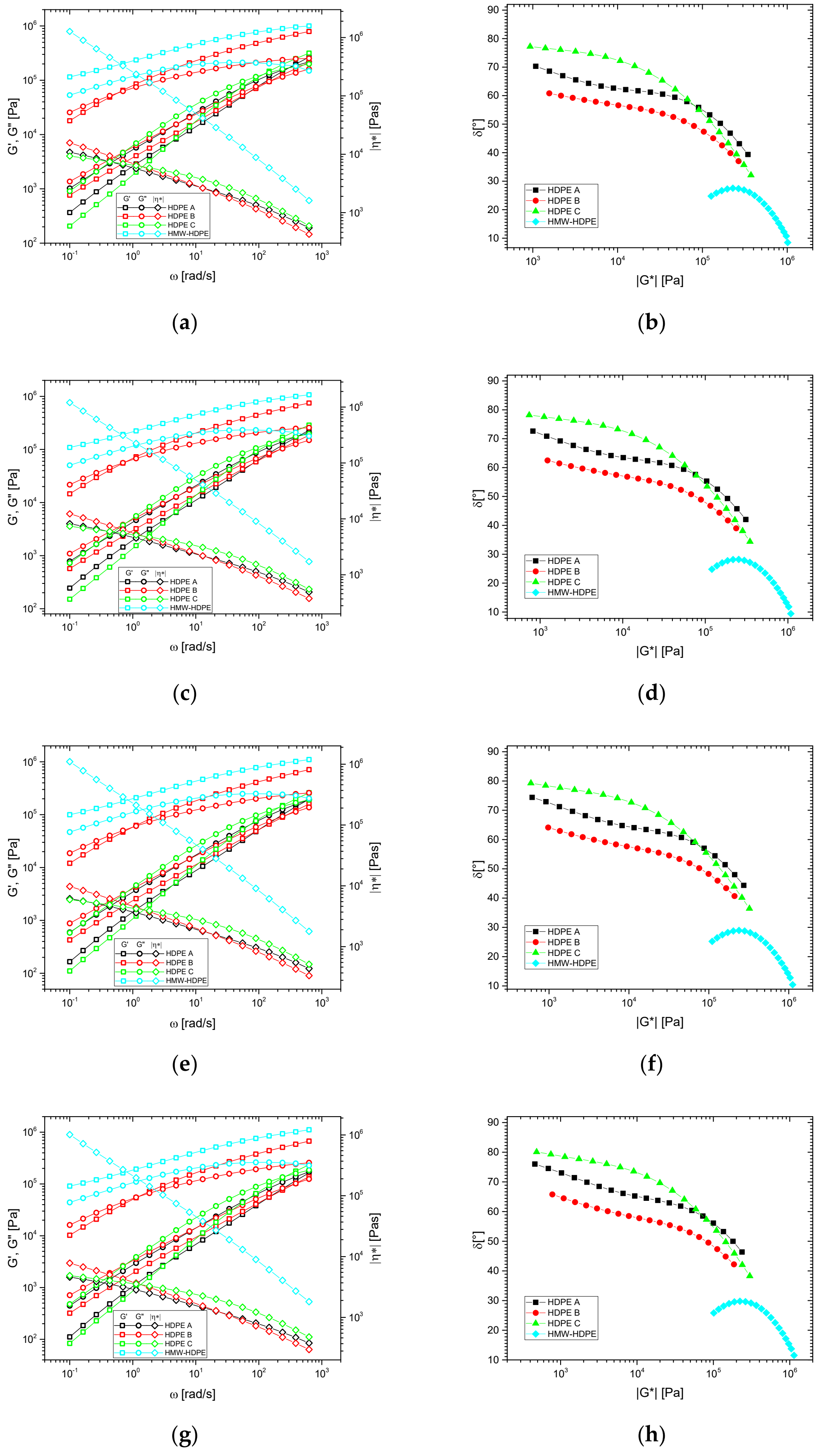

Figure 1. a) dynamic-mechanical modulus and viscosity functions of the individual blend components. T = 170 °C, $\gamma 0 \leq 5\%$. **c**) T = 190°C. **e**) T = 210 °C. **g**) T = 230 °C. **b**) corresponding δ(|G*|)-plots. T = 170 °C, $\gamma 0 \leq 5\%$. **d**) T = 190 °C. **f**) T = 210 °C. **h**) T = 230 °C.

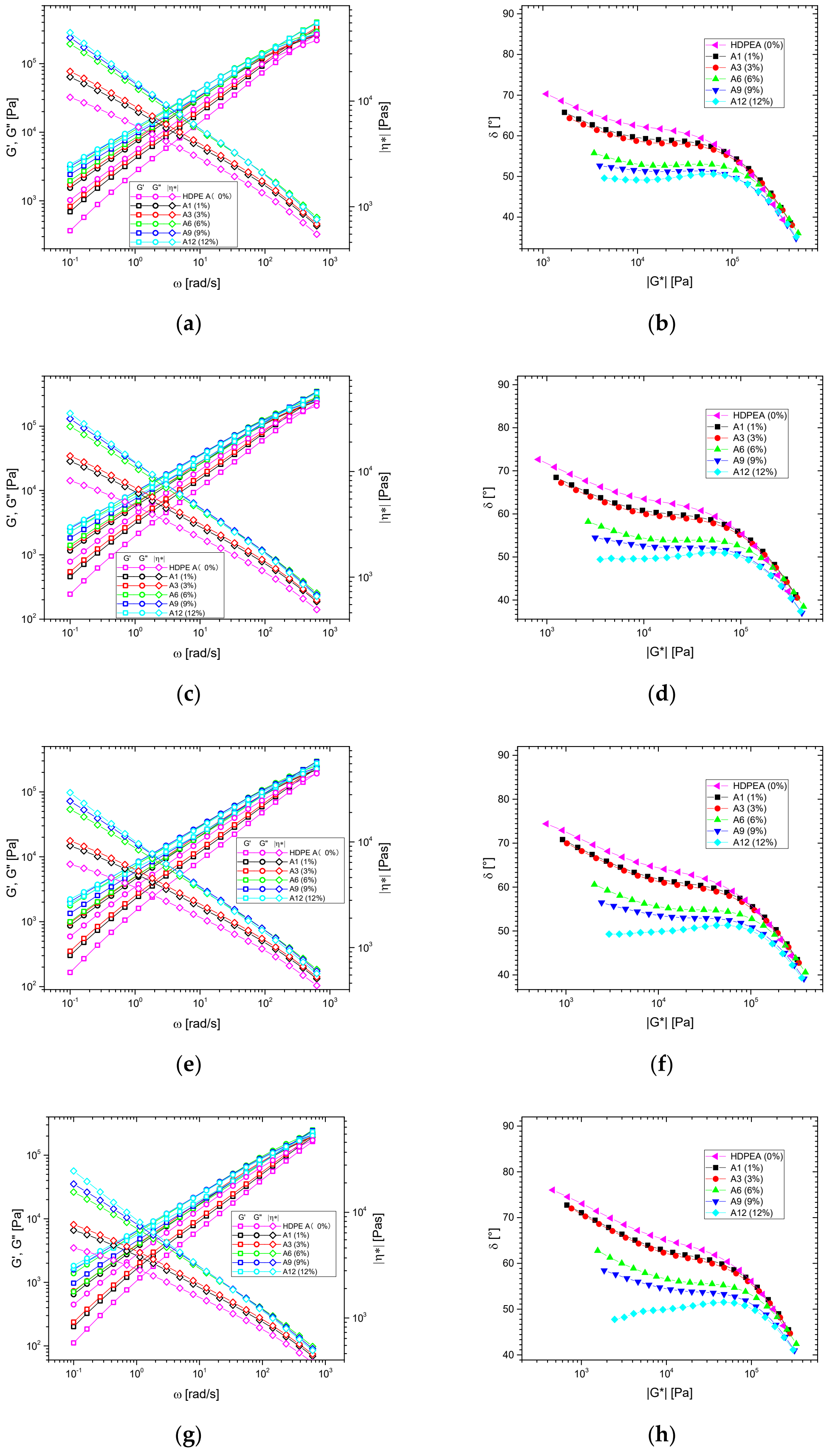

Figure 2. a) dynamic-mechanical modulus and viscosity functions of HDPE A, A1, A3, A6, A9, A12. T = 170 °C, $\gamma_0 \leq$ 5%. c) T = 190 °C. e) T = 210 °C. g) T = 230 °C. b) corresponding $\delta(|G^*|)$-plots. T = 170 °C, $\gamma_0 \leq$ 5%. d) T = 190 °C. f) T = 210 °C. h) T = 230 °C.

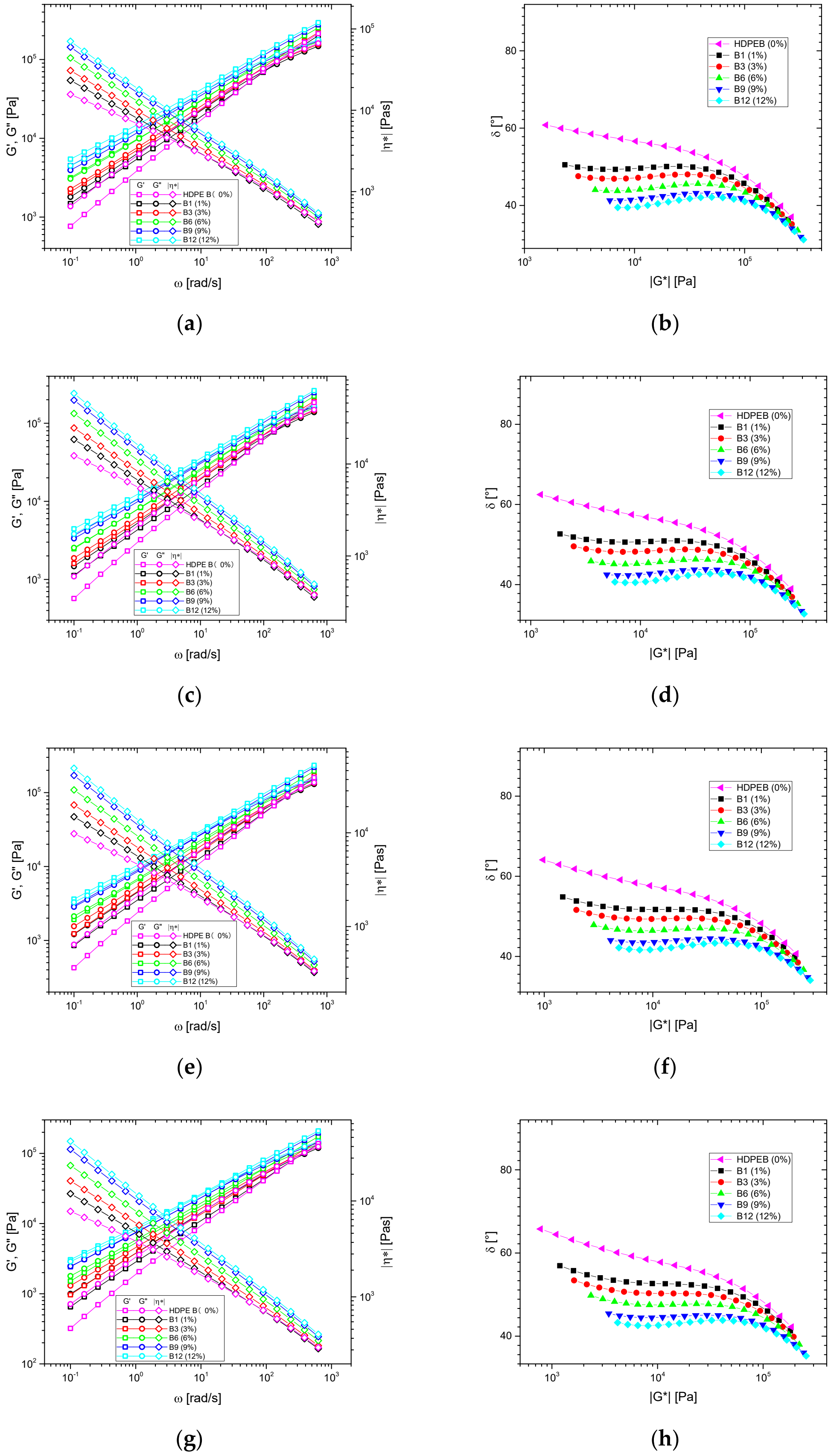

Figure 3. a) dynamic-mechanical modulus and viscosity functions of HDPE B, B1, B3, B6, B9, B12. T = 170 °C, $\gamma_0 \leq 5\%$. **c**) T = 190 °C. **e**) T = 210 °C. **g**) T = 230 °C. **b**) corresponding $\delta(|G^*|)$-plots. T = 170 °C, $\gamma_0 \leq 5\%$. **d**) T = 190 °C. **f**) T = 210 °C. **h**) T = 230 °C.

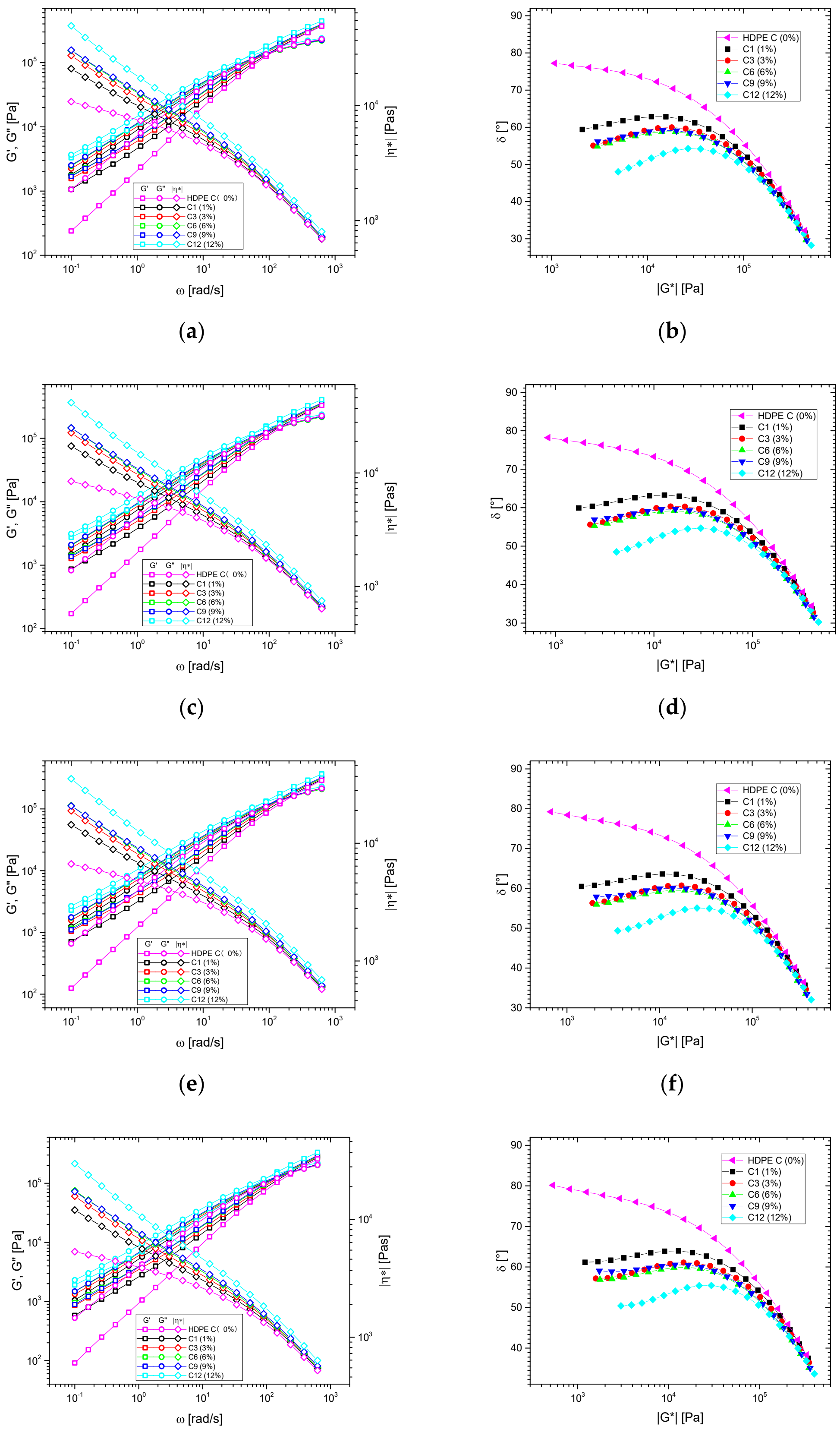

Figure 4. a) dynamic-mechanical modulus and viscosity functions of HDPE C, C1, C3, C6, C9, C12. T = 170 °C, $\gamma_0 \leq 5\%$. c) T = 190 °C. e) T = 210 °C. g) T = 230 °C. b) corresponding $\delta(|G^*|)$-plots. T = 170 °C, $\gamma_0 \leq 5\%$. d) T = 190 °C. f) T = 210 °C. h) T = 230 °C.
